# MicroRNAs as Signaling Mediators and Biomarkers of Drug- and Chemical-Induced Liver Injury

**DOI:** 10.3390/jcm4051063

**Published:** 2015-05-20

**Authors:** Mitchell R. McGill, Hartmut Jaeschke

**Affiliations:** Department of Pharmacology, Toxicology and Therapeutics, University of Kansas Medical Center, 3901 Rainbow Blvd, MS 1018, Kansas City, KS 66160, USA; E-Mail: mmcgill@kumc.edu

**Keywords:** microRNA, drug hepatotoxicity, biomarkers, acute liver failure, miR-122

## Abstract

Drug-induced liver injury (DILI) is major problem for both the drug industry and for clinicians. There are two basic categories of DILI: intrinsic and idiosyncratic. The former is the chief cause of acute liver failure in several developed countries, while the latter is the most common reason for post-marketing drug withdrawal and a major reason for failure to approve new drugs in the U.S. Although considerably more progress has been made in the study of intrinsic DILI, our understanding of both forms of drug hepatotoxicity remains incomplete. Recent work involving microRNAs (miRNAs) has advanced our knowledge of DILI in two ways: (1) possible roles of miRNAs in the pathophysiological mechanisms of DILI have been identified, and (2) circulating miRNA profiles have shown promise for the detection and diagnosis of DILI in clinical settings. The purpose of this review is to summarize major findings in these two areas of research. Taken together, exciting progress has been made in the study of miRNAs in DILI. Possible mechanisms through which miRNA species contribute to the basic mechanisms of DILI are beginning to emerge, and new miRNA-based biomarkers have the potential to greatly improve diagnosis of liver injury and prediction of patient outcomes.

## 1. Introduction

There are two major categories of drug-induced liver injury (DILI). Idiosyncratic DILI (iDILI) is unpredictable, occurs at therapeutic doses, and shows delayed toxicity that is occasionally observed in individuals following exposure of variable length to the causative agent. It occurs only rarely (<1 in 10,000 exposed individuals) and the pathophysiological mechanisms are generally poorly understood. Although drugs with higher recommended therapeutic doses tend to be associated with iDILI, it is generally regarded as non-dose-dependent [1]. In contrast, intrinsic DILI is highly predictable (strongly dose-dependent) and usually occurs within a very short timeframe (hours to days) following drug overdose. However, despite these significant differences, there are similarities. All together, DILI accounts for more cases of acute liver failure in the U.S. and several other Western countries than any other etiology [2]. Moreover, although not a single drug approved for sale in the U.S. since 1997 has been withdrawn for hepatotoxicity [3], DILI remains the most common cause for withdrawal of existing drugs from the market and for black box warnings, and is a major reason for the discontinuation of testing of new entities during pre-clinical studies [4]. Although experimental models of iDILI are notoriously poor, it is generally assumed that the findings from some studies of intrinsic DILI can translate to iDILI, particularly in the realm of biomarkers research.

MicroRNAs (miRNAs) are short (~22 nucleotide) RNA species that bind to mRNA with complementary sequences in a complex called the RNA-induced silencing complex (RISC), which prevents translation through either physical obstruction or mRNA degradation [5,6]. Thousands of miRNA species have been identified in mammalian cells. Furthermore, because some imperfect base-pairing is tolerated in RNA silencing, each miRNA can have multiple mRNA targets. Thus, the breadth of genes whose expression may be regulated in part by miRNAs and the possibilities for cross-talk between different miRNA-mediated pathways can be bewildering. Nevertheless, advances in the areas of miRNA-induced gene silencing and other possible functions or uses of miRNAs are occurring at a rapid pace.

The study of miRNAs is contributing to our understanding of DILI in two major ways. First, through basic research, we are just beginning to develop a clearer picture of the mechanisms of various forms of DILI by elucidating the roles played by miRNAs. Second, serum and urine miRNA profiles are beginning to aid in the diagnosis of DILI and in the prediction of patient outcomes. As the field progresses, improvements in our grasp of miRNA function and release will surely continue to illuminate our understanding and treatment of DILI. The purpose of this review is to summarize recent advances in the study of miRNAs in DILI, and to provide guidance for future directions. It is important to remember that many of the mechanistic data have yet to be confirmed by other groups. Nevertheless, it is clear that miRNAs are important in DILI and in hepatotoxicity due to other xenobiotics.

## 2. The microRNA Machinery

**Figure 1:**
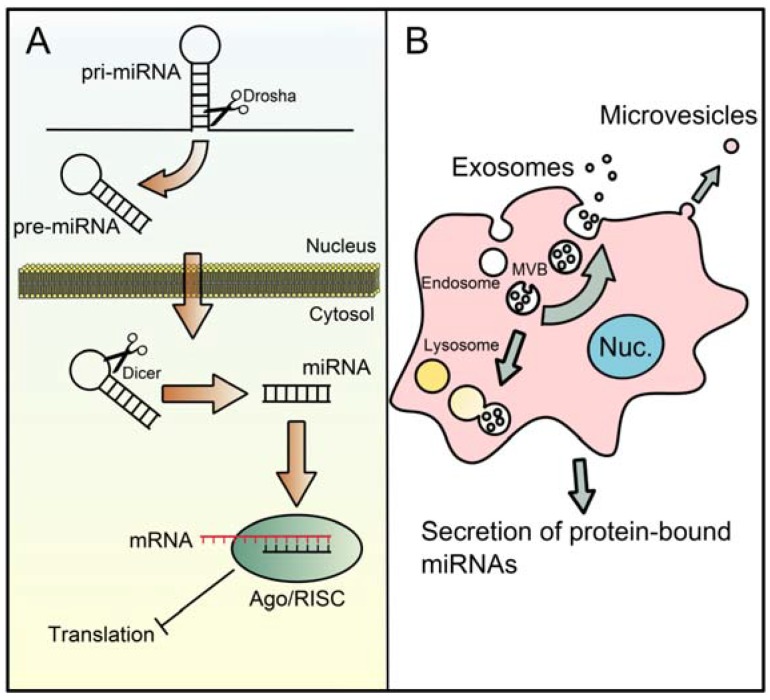
Processing and release of miRNAs. (**A**) Mature miRNAs are produced through a two-step process. First, double-stranded hairpin structures are cleaved out of long single-stranded primary miRNAs (pri-miRNA) by the Microprocessor complex that includes Drosha. This forms the pre-miRNA. Next, after the pre-miRNA is transported into the cytosol, the loop structure is removed by Dicer to yield the mature miRNA. The final miRNA is then loaded into the RNA-induced silencing complex (RISC) with an argonaute (Ago) protein. The passenger strand is removed inside the RISC and the guide strand helps to bring in the target mRNA. Translation is prevented either by physical obstruction of the mRNA or through endonuclease activity of Ago2; (**B**) Mature miRNAs can be released from cells either in exosomes, microvesicles or through secretion in a complex with proteins (namely, Ago2). Exosomes are derived from the intralumenal vesicles of multivesicular bodies (MVBs). Briefly, endosomes bud inward from the plasma membrane, and intralumenal vesicles bud inward from the endosome membrane. MVBs have two possible fates: fusion with a lysosome or fusion with the plasma membrane. The latter releases the intralumenal vesicles (now called exosomes) into the extracellular space. Microvesicles bud outward directly from the plasma membrane.

miRNA is one of three major classes of small RNA with sequences 20–30 nucleotides in length. miRNAs, in particular, begin as very long transcripts called primary miRNAs (pri-miRNA) that can be over 1000 nucleotides in length. Transcription of pri-miRNA is similar to transcription of mRNA and is mediated by RNA polymerase II [7]. Pri-miRNAs are essentially long single-stranded RNAs with a double-stranded stem-loop structure arising from within (Figure 1A). A mature miRNA is produced from a pri-miRNA through two major steps: (1) cleavage of the stem-loop structure (now called the pre-miRNA; ~65 nucleotides) off of the larger RNA sequence by the Microprocessor complex containing the RNAse Drosha; and (2) removal of the loop by the RNAse Dicer to leave only a short section of dsRNA [6,7] (Figure 1A). The first step occurs within the nucleus, while the second takes place in the cytosol after the pre-miRNA has been transported out. Export from the nucleus into the cytosol is primarily mediated by exportin-5, which recognizes a small 3' overhang on the pre-miRNA [8]. Each strand of the dsRNA is ~22 nucleotides long. One strand, then called the passenger strand, is removed when the miRNA binds with an argonaute (Ago) protein, forming the RISC, and the remaining guide strand then directs loading of the target mRNAs [7]. Translation is prevented either by physically blocking it or by stimulating degradation of the mRNA [6] (Figure 1A). Degradation may occur either through endonuclease or exonuclease activity of the Ago protein. Which strand is removed depends upon the binding properties of each [8]. Importantly, target mRNA binding is determined in large part by complementarity to nucleotides 2–7 on the 5' end of the miRNA. This is often referred to as the seed sequence [8]. Interestingly, although seed sequences can differ somewhat in different animals, there is evidence that they tend to be conserved within and across species [9]. The latter finding suggests that data from animals may be extrapolated to humans. High concentrations of miRNAs, mRNAs and miRNA machinery are found within mRNA-storage structures that are known as processing bodies (P-bodies) [8]. Interestingly, P-bodies are often seen in close association with mitochondria.

Although pri-miRNA is generally thought to be transcribed from nuclear DNA, there is limited evidence that liver mitochondria contain a distinct pool of miRNA species, as well as some of the necessary miRNA machinery to process them, and that some of these miRNAs in the mitochondria may even be produced from mitochondrial DNA [10,11]. While the exact mechanisms by which these miRNA species appear in mitochondria are not known, it is intriguing to speculate that “mitomiRs” could be important in some forms of DILI.

## 3. MicroRNAs in the Mechanisms of DILI

A number of studies have identified expression changes and both beneficial and detrimental functions of miRNAs in various models of liver injury and disease, including fatty liver [12,13,14,15], viral hepatitis [16,17,18], and obstructive cholestasis [19,20]. Although most studies of miRNA in liver injury to date have been descriptive, there is an emerging literature on the possible functions of miRNA in DILI. These studies can be grouped based on experimental approach.

*MiRNA expression studies.* Several studies have attempted to gain insight into the mechanisms of hepatotoxicity of xenobiotics by measuring changes in miRNA expression in the liver after treatment with these compounds. These experiments have yielded some interesting results.

One of the first studies to examine changes in liver miRNA expression during DILI found that several of the same miRNAs either increased or decreased ≥1.5-fold after treatment with acetaminophen (APAP) or carbon tetrachloride (CCl_4_) [21]. Although CCl_4_ is not a drug, it does cause acute liver injury similar to APAP and other drugs, and is a useful general model of xenobiotic hepatotoxicity. Two miRNAs (miR-298 and -370), in particular, decreased at early time points after APAP treatment either *in vivo* or *in vitro*. The authors concluded that suppression of these two miRNAs likely contributes to APAP hepatotoxicity because they are thought to target antioxidant genes [21]. Interestingly, a similar study found that some miRNA changes in the liver were zone specific [22]. Unfortunately, a major drawback of these two studies was the use of rats, which are generally resistant to APAP toxicity and are known to be a poor model of APAP-induced liver injury [23]. Indeed, although the authors of the earlier study reported that there was evidence of necrosis in liver tissue sections [21], no histology was shown in the paper and clinical biochemistry such as serum ALT was not reported. Thus, the results of these two studies must be interpreted with caution. Nevertheless, this earlier work did provide a proof-of-principle for APAP-induced changes in miRNA levels that was later confirmed in mice [24], a more relevant model of APAP toxicity in humans [23,25,26].

While APAP and CCl_4_ are classic models of intrinsic DILI, halothane may be considered closer to an idiosyncratic hepatotoxicant. In some humans, halothane causes otherwise asymptomatic increases in serum liver injury markers, and in a few such cases the injury becomes much more severe upon later re-exposure. The toxicity begins with the formation of the reactive metabolite trifluoroacetylchloride. Although this metabolite is necessary for the injury, it is not sufficient and variation in drug metabolism does not explain variation in toxicity [27,28]. There is evidence that, similar to many idiosyncratic toxicants, the immune system plays a role in halothane-induced liver injury [29,30,31]. Specifically, innate immunity may be important in the initial stages of the injury, while humoral immunity becomes important later [31]. Interestingly, it was recently reported that the hepatic concentrations of a number of miRNAs that may be involved in inflammatory signaling are altered early after halothane treatment in mice [32]. In particular, one miRNA (miR-106b) that is thought to regulate Stat3 activation was decreased [32]. Stat3 had previously been shown to be important for differentiation of Th17 cells, which can produce pro-inflammatory cytokines [32]. These data may suggest that miRNAs are important in the mechanisms of some cases of iDILI. In fact, it is tempting to speculate that variation in miRNA expression in humans could explain some cases of iDILI.

MicroRNA expression changes have also been observed in the liver damage caused by several environmental hepatotoxicants. Like CCl_4_, these are not drugs, but they can be useful for our understanding of general xenobiotic-induced hepatotoxicity. For example, one study found that the expression of several hepatic miRNAs was changed in mice after treatment with dioxin but before the onset of liver injury, suggesting that these miRNAs may be involved in the initiation of toxicity [33]. In particular, miR-101a was reduced. At the same time, the expression of certain pro-inflammatory mediators was increased. The authors concluded that the decrease in miR-101a leads to greater liver injury through increased production of Cox2 [33]. In another study, numerous miRNAs were found to be differentially expressed between control mice and mice treated with the hepatocarcinogen aflatoxin B1 [34].

MicroRNA expression profiles change not only during liver injury, but also during liver regeneration. It has been shown that miRNA levels change during partial hepatectomy, and that these changes can be modulated by ethanol feeding [35,36]. MiR-21 has been shown to promote cell proliferation and tumor growth, in part by inhibiting expression of the tumor suppressor gene phosphatase and tensin homolog (PTEN) and altering expression of matrix metalloproteinases [37]. Greater induction of miR-21 was observed in ethanol-fed rats undergoing partial hepatectomy than in pair fed control animals. Interestingly, ethanol feeding also impairs liver regeneration; the authors of the study speculated that miR-21 may be responsible for this effect [35]. Induction of miR-21 during liver regeneration was confirmed in a later study [38].

Overall, miRNA expression studies have yielded interesting hypotheses concerning the mechanisms of DILI and recovery afterword. These data strongly suggest that miRNA species are important in regulating several aspects of hepatotoxicity and regeneration. Of course, it is important to keep in mind that any study of large scale gene expression changes may be limited by the methodology (e.g., sequencing, microarrays, PCR, *etc.*) used to measure those changes. Different approaches may identify different miRNAs. Future studies may obtain results that differ from these results. Furthermore, the descriptive nature of these studies also limits their usefulness. Fortunately, a few more detailed mechanistic studies are available that also support the importance of miRNAs in DILI.

*Mechanistic miRNA studies*. Several studies have more directly investigated possible roles of miRNAs in hepatotoxicity using specific interventions either in cultured cells or in animal models. One group found that expression of miRNA-491-5p is increased in mice after treatment with a Fas receptor agonist and that overexpression of this miRNA in HepG2 cells sensitizes them to TNFα-induced liver cell injury through decreased NFκB activity and decreased levels of pro-survival proteins [39]. In another example, it was shown that let-7 miRNA family members regulate expression of the co-repressor protein Bach1 that normally reduces production of the antioxidant enzyme heme oxygenase-1 [40]. Consistent with this, a let-7b mimic was found to protect against oxidative stress-induced cell death in the hepatic cell line Huh7 [40]. A few other studies using hepatoma cells and/or primary hepatocytes have shown other interesting findings concerning the roles of miRNAs in hepatocyte injury [20,41,42]. It was reported in one example that miR-199a-5p protects against bile acid-induced hepatocyte damage by reducing expression of ER stress signaling proteins [20]. Another group found that miR-214 is induced by ethanol exposure in both rats and cultured cells and that it targets glutathione reductase and cytochrome P450 oxidoreductase, thereby increasing oxidative stress and injury [41]. It has also been shown that oxidative stress itself, frequently caused by hepatotoxicants, can activate the cell death protein Rab38 in L02 cells, and that miR-124 targets Rab38 and reduces its expression [42].

*In vivo*, it was shown that estradiol can induce expression of miR-29 in the liver and reduce evidence of fibrosis after CCl_4_ treatment in mice [43]. Importantly, overexpression of miR-29 also reduced collagen deposition in this model [43]. The authors concluded that miR-29 may be responsible for the resistance of female mice to CCl_4_-induced liver injury [43]. A similar study revealed an increase in miR-21 in hepatic stellate cells (HSCs) in both humans with cirrhosis and in the CCl_4_ murine fibrosis model [44]. Interestingly, miR-21 was found to activate HSCs and knockdown of miR-21 reduced evidence of fibrosis in the mice [44]. The mechanism of protection appeared to involve a complex signaling loop [44]. It is interesting to note that miR-21, as previously discussed, is also a pro-regenerative signal in the liver, illustrating the pleiotropic effects of miRNAs. More recently, another group found that miR-122, the most abundant miRNA species in the liver, is decreased in the acute CCl_4_ and thioacetamide models of hepatotoxicity and that this appeared to be due to decreased transcription of its pri-miRNA [45]. The authors speculated that this is part of a liver stress response [45].

All together, it is clear that miRNAs can have important roles in the mechanisms of hepatotoxicity caused by drugs and other xenobiotics. It seems that some miRNAs have beneficial, protective effects, while others contribute to the injury. Additional work will improve our understanding of the most common miRNAs involved in DILI. Several specific examples of proposed effects of miRNAs in liver injury are given in Table 1.

**Table 1:** Examples of the possible effects of various miRNAs in the liver.

Study Type	Effect	Putative miRNA(s) Involved	References
*In vivo*	Liver injury	Decrease: miR-101a, -106b, -298, -370, -491-5p miR-122?	[21,32,33,39,45]
	Fibrosis	Decrease: miR-29 Increase: miR-21	[43] [44]
	Liver regeneration	Pro-regenerative: miR-21	[35,36,38]
*In vitro*	Cell injury	Decrease: Let-7b, miR-124	[40]
	Cell proliferation	Pro-proliferative: miR-21	[44]

Specific examples of proposed effects of various miRNAs in hepatotoxicity based on whether the miRNA levels increase or decrease during the observed effect and on the basis of additional mechanistic studies.

In addition to their functions in the pathophysiological mechanisms of hepatocyte damage, there is some indication that certain miRNA species are involved in dedifferentiation and proliferation of isolated hepatocytes *in vitro* [46]. It is possible that these miRNAs could be targeted in primary hepatocytes in order to maintain hepatic phenotype for use in long term drug toxicity studies [46]. The latter could be a major advance in the field of *in vitro* drug toxicity.

## 4. MicroRNAs as Biomarkers of DILI

The first study to report the detection of miRNA in biological fluids was published nearly a decade ago [47] and this area of research has rapidly grown since then. Considerable information is now available concerning the potential utility of circulating miRNAs as biomarkers and the mechanisms of miRNA release.

*Biomarkers for diagnosis and prognosis.* Numerous studies have shown that serum miRNA concentrations dramatically change during liver injury [24,48,49,50,51,52,53,54,55,56,57]. Wang *et al.* [24] were the first to demonstrate this effect in drug hepatotoxicity. They found that miR-122 and miR-192 increased in the circulation of mice after treatment with toxic doses of APAP [24]. These findings were soon confirmed in APAP overdose patients [49]. Several other miRNAs (e.g., miR-155, miR-125b, and miR-146a) have also been shown to increase in serum from mice after APAP treatment [24,58]. More recently, several screening studies have shown that the entire serum profile of miRNAs undergoes large-scale changes during APAP-induced liver injury in humans [53,59,60,61]. For reference, a list of miRNAs that are most commonly reported as increased in serum from APAP overdose patients and mice is shown in Table 2. Increases are most often seen in miR-122, miR-192 and miR-125b levels (Table 2). Relatively few miRNA decreases in serum have been observed. Interestingly, changes in urine miRNA levels have also been reported during drug hepatotoxicity, though the mechanisms and significance of this are less well-studied [61].

There is some evidence that serum miRNAs may be useful for diagnosis of liver injury and for prediction of patient outcome. miR-122 has been shown to be a very early and sensitive biomarker of DILI. The earliest studies of serum miRNA in APAP hepatotoxicity found that even subtoxic doses of APAP could increase miR-122 and miR-192 levels in serum of mice while ALT was unaltered [24]. Similarly, it was reported in a particularly interesting case study that miR-122 levels were elevated in a very early-presenting patient who lacked any other clinical evidence of liver damage [62]. The patient returned to the hospital two days later with elevated ALT and evidence of coagulopathy [62]. These data suggest that serum miR-122 measurement may be useful during early phase human trials to identify drugs that cause subclinical liver cell stress and therefore have the potential to cause hepatotoxicity (including idiosyncratic hepatotoxicity) once on the market. Importantly, serum miRNA profiles may be even more useful than individual miRNAs for diagnosis. Recent work has shown that miRNA profiles can actually differentiate between cases of acute liver injury due to different etiologies [53]. Several miRNAs were found to discriminate between APAP hepatotoxicity and ischemic hepatitis in humans despite similar release of liver enzymes [53]. Combined with the fact that the mechanism of release also differs with etiology [58], there is obvious potential for the clinical use of miRNAs to differentiate between specific causes of liver injury and make more accurate diagnoses.

**Table 2:** miRNAs elevated in serum during acetaminophen hepatotoxicity.

Human Studies (hsa-)			Human (hsa-) and Mouse (mur-) ^d^
Exact, All Studies ^a^	Exact, Adult Studies ^b^	Similar, Adult and Child Studies ^c^	
**miR-122**	**miR-122**	miR-193a/b-5p	**miR-122**
	**miR-125b-5p**	miR-378a/b/c/e/g/h/i	**miR-192**
	miR-483-5p	miR-30a/d-5p	**miR-125b**
			miR-202-3p

Data are derived from Wang *et al.* [24]; Bala *et al.* [58]; Ward *et al.* [59]; Ward *et al.* [53]; Krauskopf *et al.* [60]; and Yang *et al.* [56]. ^a^ Exact miRNA is reported in all human studies; ^b^ Exact miRNA is reported in all adult human studies; ^c^ Similar miRNAs reported in child study and at least one adult study. In miRNA nomenclature, different letters following miRNA numbers denote similar but non-identical sequences from different genomic loci; ^d^ Exact miRNA reported in at least one rodent study and at least one human study. The most commonly reported miRNAs overall are listed in bold.

Emerging data also suggest that serum miRNAs can be useful for patient prognosis. Starkey Lewis *et al.* [49] found that the serum levels of miR-122 from day 1 of study enrollment were higher in patients who met the King’s College Criteria for liver transplantation, although the data did not achieve significance. It was later shown that admission levels of miR-122 could predict later development of hepatotoxicity in a similar study population [63]. Taken together, these data suggest a very promising future for the clinical use of miRNA biomarkers to predict patient outcomes after liver injury.

Currently, alanine aminotransferase (ALT) and aspartate aminotransferase (AST) are the most common serum biomarkers of liver injury. Although other biomarkers are also used, such as serum bilirubin, they are generally considered indicators of liver function and only increase after injury has progressed to late stages. A problem with both ALT and AST is that they do not seem to increase until after hepatocellular death has begun, so they can be used to detect injury but not to predict developing injury. Another issue is that aminotransferases, especially ALT, are generally poor predictors of patient death in DILI [64,65,66]. Finally, ALT and AST increase non-discriminately in liver injury; they cannot be used to determine etiology except in a few cases in which the AST/ALT ratio is useful. Thus, miRNAs may be better for clinical use in the future than the currently accepted biomarkers. Moreover, routine measurement of serum miRNAs, such as miR-122, during early clinical testing may allow detection of drugs with the potential for idiosyncratic DILI before they can be approved. Measurement of ALT and bilirubin together (a variation of Hy’s law) has already helped to dramatically reduce the number of hepatotoxic drugs that reach the market [3], and measuring very sensitive miRNAs during early trials may further improve drug safety evaluation. For example, there is evidence that miR-122 can increase without a concurrent increase in ALT [62]. Thus, it is possible that drugs with the potential to cause hepatotoxicity could be identified, even if they do not cause large changes in ALT levels during clinical trials.

*Mechanisms of release*. Around the time that miRNA was first measured in circulation [47], another study found that exosomes in the media of cultured cells contain miRNA [67]. This led to the hypothesis that much circulating miRNA is packaged within extracellular vesicles, namely exosomes and microvesicles. Generally, exosomes are small (50–100 nm in diameter) lipid vesicles derived from intraluminal vesicles (ILVs) of endosomes [68]. ILV-containing endosomes are often called multivesicular bodies (MVBs). ILVs are formed by inward budding of the endosomal membrane into the lumen of the endosome [68,69] (Figure 1B). Once formed, a MVB has two possible fates: fusion with a lysosome and degradation of the endosome contents, or fusion with the cell membrane and release of ILVs—now called exosomes—into circulation [68,69] (Figure 1B). The exact mechanisms of plasma membrane fusion and exosome release are still being investigated [69]. Microvesicles, on the other hand, are larger (100–1000 nm in diameter) and actually bud off of the plasma membrane itself [69] (Figure 1B). The mechanisms of microvesicle shedding are complex and less well-studied. Although extracellular vesicles are an important route for release of miRNA into circulation, most circulating miRNA is actually found outside of exosomes and microvesicles [70,71]. By far, the majority of miRNAs in blood are protein-bound (often associated with Ago2) and do not have the benefit of a membrane-enclosure (Figure 1B).

The mechanisms of miRNA release from the liver during hepatotoxicity vary depending upon etiology. Bala *et al.* [58] found that most miR-122 and miR-155 in plasma was associated with exosome-rich fractions rather than protein-rich supernatants in a mouse model of alcoholic liver disease with minor ALT elevations, while the opposite was seen in mice with APAP hepatotoxicity that caused very high ALT levels. Moreover, the amounts of these miRNAs associated with exosomes instead of soluble proteins appeared to decrease somewhat at later time points after APAP. Unfortunately, the authors did not specifically evaluate miRNA levels in microvesicles. Nevertheless, their findings may suggest that exosomes play an important role in early release of miRNAs during DILI, while continued release at later time points is more likely due to cell death and loss of plasma membrane integrity. One caveat of the latter idea is that several groups have reported increases in miRNAs that are not thought to be abundant in normal liver, or are thought to be specific for other tissues, during APAP-induced liver injury in both mice and humans [24,53,60]. While it is possible that hepatocytes have increased expression of various miRNAs that are not normally associated with the liver during DILI, it has been proposed that these other non-liver miRNAs indicate APAP-induced stress or injury in other tissues [24,53,60].

While there is evidence that some circulating miRNAs are important for intercellular communication among many different tissues and cell types [68,72], it has been suggested that most are simply waste released by healthy or dying cells during normal physiological processes and cell turnover [71]. Moreover, recent work has indicated that some circulating miRNAs are actually derived from gut flora [73]. However, regardless of origin or function, it is clear that circulating miRNAs may be useful biomarkers in the study and treatment of DILI.

*Technical issues with miRNA biomarkers.* As with tissue miRNA expression changes, it is critical to consider the strengths and weaknesses of different methodologies used for miRNA detection and quantification [55]. miRNA research is still a growing field; technical issues are still being worked out and new miRNAs are still being discovered [74,75]. There are numerous methods available for both the isolation (e.g., liquid extraction, RNA-binding columns, *etc.*) and measurement (qPCR, RNA sequencing, microarray, immunoassay, *etc.*) of circulating miRNAs, and different methods are likely to yield somewhat different results. For example, while several groups have reported an increase in miR-192 during APAP hepatotoxicity in the circulation of both mice and humans [24,49,60], at least one was unable to detect a significant increase in this miRNA in samples from APAP overdose patients [53]. Issues of data normalization have also been discussed in the DILI literature [76]. Moreover, hemolysis can also affect miRNA levels in serum [77,78]. Clearly, it is important to consider what sample preparation, measurement and normalization procedures are appropriate for a given miRNA experiment. Several reviews are available on this subject [55,74,75].

## 5. Conclusions

Although the physiological and pathophysiological roles of miRNAs in DILI are only beginning to be understood, it is clear that this is an important area of research. Emerging data suggest that miRNAs have both positive and negative roles in the mechanisms of drug hepatotoxicity. Importantly, there is also strong evidence that certain miRNAs in serum, like miR-122, as well as profiles of serum miRNAs, may be clinically useful for both diagnosis of liver injury and prediction of patient outcome. There is clear evidence that miR-122 is a very sensitive and early indicator of liver damage, which may make it useful during early drug trials for the identification of drugs with the potential to cause liver injury. Moreover, it may be possible to diagnose specific etiologies of liver injury using miRNA profiles, and even to predict the need for liver transplantation. The future of miRNA research in DILI looks bright.
